# A novel cancer-associated lncRNA, LINC01123, participates in tumor progression, metabolism, immune escape, and resistance

**DOI:** 10.3389/fimmu.2025.1480447

**Published:** 2025-04-04

**Authors:** Qiang Liu, He Huang, Shuwen Zhang, Fangteng Liu, Ting Lou

**Affiliations:** ^1^ Department of General Surgery, Jiujiang Hospital of Traditional Chinese Medicine, Jiujiang, Jiangxi, China; ^2^ Department of Gastrointestinal Surgery, The Second Affiliated Hospital, Jiangxi Medical College, Nanchang University, Nanchang, Jiangxi, China; ^3^ Queen Mary College, Nanchang University, Nanchang, Jiangxi, China; ^4^ Department of Hospital Admission and Medical Record Management, The Second Medical Center & National Clinical Research Center for Geriatric Diseases, Chinese People's Liberation Army (PLA) General Hospital, Beijing, China

**Keywords:** LINC01123, human tumors, biological functions, regulatory mechanism, cancer biomarker

## Abstract

Long Intergenic Non-Protein Coding RNA 1123 (LINC01123), located on human chromosome 2q13, is a pivotal factor in tumorigenesis, exerting multifaceted oncogenic effects. Its expression strongly correlates with clinicopathological features, patient survival, and disease progression. *In vivo* and *in vitro* experiments further demonstrate that LINC01123 influences diverse cellular processes, including proliferation, apoptosis, viability, migration, invasion, stemness, and tumor growth. Notably, it also regulates metabolic reprogramming, immune escape, and tumor cell resistance to treatment. LINC01123 is regulated by multiple transcription factors and participates in gene regulation through protein interactions and competitive endogenous RNA (ceRNA) networks, thereby modulating cancer-promoting effects. This work systematically elucidates its primary functions and molecular mechanisms driving cancer initiation and progression, suggesting that LINC01123 might serve as a novel potential oncogenic driver and biomarker in various cancers.

## 1 Introduction

Long non-coding RNAs (lncRNAs) are RNA molecules longer than 200 nucleotides that do not encode proteins (1–4). Initially considered “junk” RNA, they are now recognized as crucial players in human diseases (5–7), particularly cancer (8–10). LncRNAs are classified based on their proximity to protein-coding genes, including sense, antisense, intronic, bidirectional, or intergenic types (11–13). Mechanistically, one of the main functions of lncRNAs involves the ceRNA network (14–16). Acting as “sponges” for microRNAs (miRNAs), lncRNAs bind to miRNAs and modulate their availability for target mRNAs, thereby indirectly regulating gene expression. Disruptions in the ceRNA network involving lncRNAs are evident in various cancers (17–19). Accumulating evidence underscores the pivotal role of lncRNAs in cancer development and progression, highlighting their potential as targets for innovative therapeutic strategies (20–22).

*Homo sapiens* (human) Long Intergenic Non-Protein Coding RNA 1123 (LINC01123) is classified as a lncRNA gene located on chromosome 2q13. Spanning a length of 8302 nucleotides (nt), this gene comprises four exons (https://www.ncbi.nlm.nih.gov/gene/440894). The LINC01123 gene produces two splice variants: ENST00000419296.1, which is 2436 base pairs (bp) long, and ENST00000336905.3, which spans 2271 bp (Source: https://www.ensembl.org/Homo_sapiens/Gene/Summary?g=ENSG00000204588;r=2:109987063-109996140). LINC01123 has recently emerged as a key player in the pathogenesis of various diseases, including deep vein thrombosis (23), atherosclerosis (AS) (24), and acute cerebral infarction (25). Notably, its role in cancer progression has sparked considerable interest (26–28). LINC01123 is upregulated in a wide array of human tumors, and elevated expression levels of LINC01123 in cancerous samples correlate with adverse clinicopathological features and poor prognosis, including lymph node metastasis, tumor size, clinical stage, overall survival (OS), disease-free survival (DFS), and biochemical recurrence (BCR)-free survival. Furthermore, LINC01123 plays a critical role in crucial biological processes such as epithelial-mesenchymal transition (EMT), tumor cell growth, and invasion. Given its pivotal role in tumor progression, LINC01123 is expected to serve as a valuable tumor biomarker and contribute to the development of effective therapeutic strategies across various malignancies.

In this work, we provide a comprehensive summary of the latest research on the roles of LINC01123 in tumor development. We focus on LINC01123 expression patterns, associated clinical characteristics, its potential as a cancer biomarker, and its biological functions in tumor progression. Additionally, we examine the underlying mechanisms driving LINC01123’s effects in various malignancies. This review highlights the promising prospects of LINC01123 as a target for therapeutic interventions in different cancer types.

## 2 Expression and clinical significance of LINC01123 in human tumors

Recently identified as an oncogenic lncRNA, LINC01123 demonstrates significant upregulation across multiple cancer types originating from various organ systems (26–37). These include cancers of the nervous system (glioma) (29), respiratory system (lung cancer, LC) (28, 30), head and neck region (oral squamous cell carcinoma, OSCC; head and neck squamous cell carcinoma, HNSCC) (26, 27), digestive system (hepatocellular carcinoma, HCC; colorectal cancer, CRC; intrahepatic cholangiocarcinoma) (31–35), and reproductive system (ovarian cancer, OC; cervical cancer, CC) (36, 37), as summarized in **Table 1**.

**Table 1:** Relationship between LINC01123 expression in tumor samples and clinicopathological features and prognosis in cancer patients.

Cancer type	Expression	Significant clinical variables	End-points	Unfavorable	Ref.
Colorectal cancer	Up-regulated	TNM stage, lymph-node metastasis	OS, DFS	High expression	(32)
Non-small cell lung cancer	Up-regulated	TNM stages, T stage, lymph node metastasis, mortality	OS	High expression	(28)
Hepatocellular carcinoma	Up-regulated	Tumor size, venous infiltration, TNM stage	OS	High expression	(31)
Oral squamous cell carcinoma	Up-regulated	Lymph node metastasis, TNM stage	OS	High expression	(26)
Colorectal cancer	Up-regulated	–	OS	High expression	(33)
Head and neck squamous cell carcinoma	Up-regulated	–	OS	High expression	(27)
Prostate cancer	–	–	BCR-free survival	High expression	(38)
Cervical cancer	Up-regulated	Lymph node metastasis, FIGO stage	–	–	(37)
Colon cancer	Up-regulated	–	–	–	(34)
Intrahepatic cholangiocarcinoma	Up-regulated	–	–	–	(35)
Ovarian cancer	Up-regulated	–	–	–	(36)
Glioma	Up-regulated	–	–	–	(29)
Lung adenocarcinoma	Up-regulated	–	–	–	(30)

TNM, Tumor; Node, Metastasis; FIGO, International Federation of Gynecology and Obstetrics; OS, Overall Survival; DFS, Disease-Free Survival; BCR, Biochemical Recurrence.

Research has investigated the relationship between LINC01123 expression and clinicopathological features across various cancer types (see **Table 1**). In OSCC, elevated expression of LINC01123 is associated with lymph node metastasis and advanced TNM stage (26). In non-small cell lung cancer (NSCLC), LINC01123 expression is significantly related to TNM stages, T stage, and lymph node metastasis (28). In HCC, increased levels of LINC01123 indicate larger tumor size, venous infiltration, and advanced TNM stage (31). In CRC, higher LINC01123 expression is observed in patients with advanced TNM stage and lymph node metastasis (32). Similarly, in CC, LINC01123 shows a significant positive correlation with lymph node metastasis and higher International Federation of Gynecology and Obstetrics (FIGO) stage (37). Additionally, high LINC01123 expression correlates highly with shorter OS in patients with OSCC (26), HNSCC (27), NSCLC (28), and HCC (31). And high LINC01123 expression levels are associated with worse OS and DFS in CRC patients (32), and LINC01123 is negatively associated with BCR-free survival in prostate cancer (38).

## 3 Upstream regulation of LINC01123 by trans-acting factors

**Figure 1:**
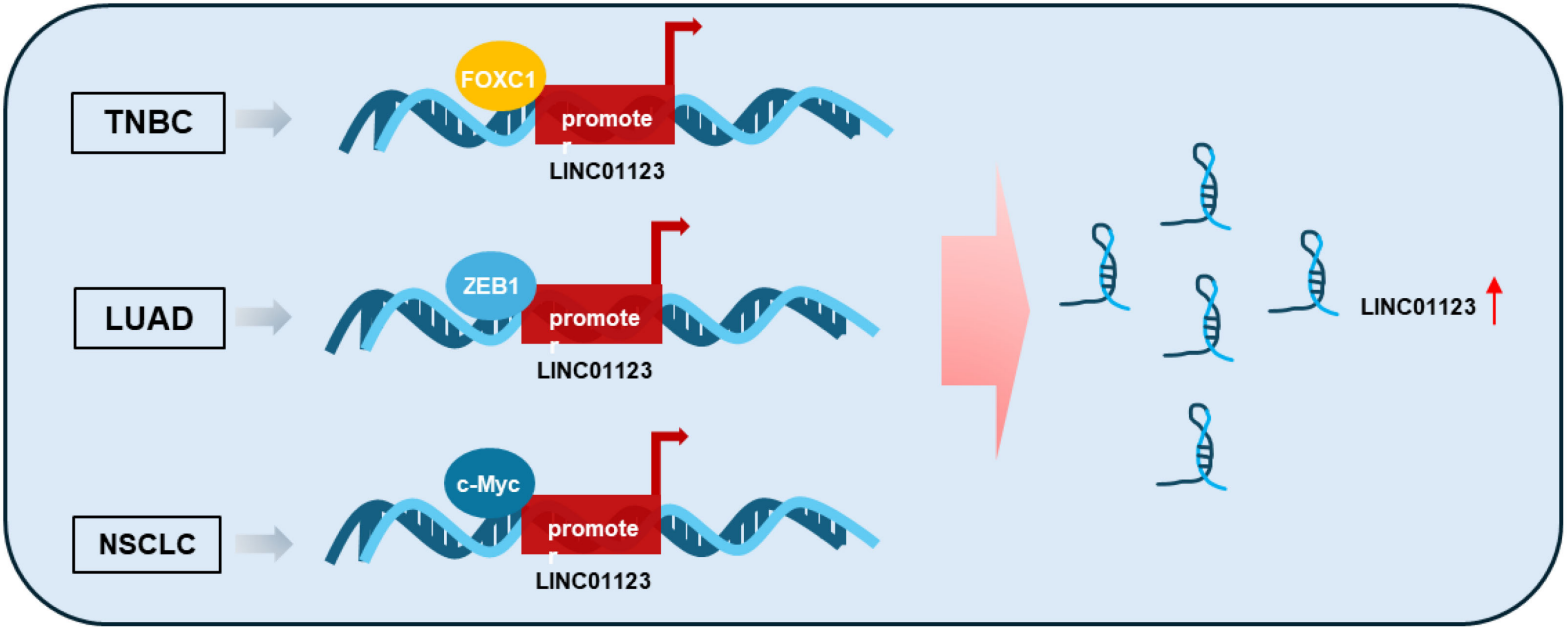
Mechanism by which the expression of LINC01123 is increased in tumors. Specific transcription factors such as FOXC1, ZEB1, and c-Myc bind to the promoter region of LINC01123, thereby initiating its transcription. TNBC, Triple-Negative Breast Cancer; LUAD, Lung Adenocarcinoma; NSCLC, Non-Small Cell Lung Cancer.

Research has shown that the expression of LINC01123 is regulated by multiple key transcription factors across various tumors (**Figure 1**) (28, 39, 40). In triple-negative breast cancer (TNBC), LINC01123 is transcriptionally amplified by FOXC1, which directly binds to the LINC01123 promoter and promotes malignant cellular processes in tumor cells (39). This regulatory mechanism suggests that targeting FOXC1 or its interaction with LINC01123 could potentially disrupt tumor progression in TNBC. In lung cancer, studies demonstrate that ZEB1 binds to the LINC01123 promoter regions, initiating transcription and upregulating LINC01123 (40). This upregulation enhances malignant functions in lung adenocarcinoma (LUAD) cells through the miR-449b-5p/NOTCH1 axis, highlighting the potential of targeting this pathway to suppress LUAD progression. Additionally, the transcription factor c-Myc also induces LINC01123 expression, functioning as an oncogene by promoting proliferation and aerobic glycolysis in NSCLC through the miR-199a-5p/c-Myc feedback loop (28). These findings collectively underscore the critical role of transcription factors in regulating LINC01123 and suggest that disrupting these regulatory networks could offer novel therapeutic strategies for multiple cancer types.

## 4 Oncogenic roles of LINC01123 in tumorigenesis and development

Recent studies have extensively examined the role of LINC01123 across eleven different types of tumors, including glioma (29), OSCC (27), HNSCC (27), TNBC (39), NSCLC (28), LUAD (30, 40, 41), HCC (31), CRC (32–34, 42), OC (36), CC (37) and osteosarcoma (43). These investigations highlight the multifaceted role of LINC01123, as evidenced by various *in vivo* and *in vitro* experiments summarized in **Table 2**.

**Table 2:** The functions and regulatory mechanisms of LINC01123 in human cancers.

Cancer type	Experiments	Cellular functions	Related molecule/pathway	Role	Ref.
Glioma	*In vitro* and *in vivo*	Cell proliferation, apoptosis, radioresistance, tumor growth	LINC01123/miR-151a/CENPB	Cancer-promoting	(29)
Oral squamous cell carcinoma	*In vitro*	Cell proliferation, migration, invasion	LINC01123/miR-34a-5p	Cancer-promoting	(26)
Head and neck squamous cell carcinoma	*In vitro* and *in vivo*	Cell proliferation, migration, and invasion, immune escape, tumor growth	LINC01123/miR-214-3p/B7–H3	Cancer-promoting	(27)
Triple negative breast cancer	*In vitro*	Cell proliferation, cell apoptosis, cell viability	LINC01123/miR-663a/CMIP	Cancer-promoting	(39)
Lung adenocarcinoma	–	Cisplatin resistance	LINC01123/hsa−miR−152−3p/MED12, LINC01123/hsa−miR−152−3P/NCAM1, LINC01123/hsa−miR−152−3P/ARF4, LINC01123/hsa−miR−762/NCAM1, LINC01123/hsa−miR−762/RHBG	Cancer-promoting	(41)
Lung adenocarcinoma	*In vitro* and *in vivo*	Cell proliferation, migration, tumor growth	LINC01123/miR-4766-5p/PYCR1	Cancer-promoting	(30)
Non-small cell lung cancer	*In vitro* and *in vivo*	Cell proliferation, metabolic reprogramming(glycolysis), tumor growth	LINC01123/miR-199a-5p/c-Myc	Cancer-promoting	(28)
Lung adenocarcinoma	*In vitro*	Cell proliferation, apoptosis, migration, EMT, stemness	LINC01123/miR-449b-5p/NOTCH1, NOTCH signaling pathway	Cancer-promoting	(40)
Hepatocellular carcinoma	*In vitro* and *in vivo*	Cell proliferation, migration, invasion, tumor growth	LINC01123/miR-34a-5p/TUFT1	Cancer-promoting	(31)
Colorectal cancer	*In vitro*	Cell proliferation, invasion	LINC01123/miR-625-5p/LASP1	Cancer-promoting	(32)
Colorectal cancer	*In vitro* and *in vivo*	Cell proliferation, migration, invasion, tumor growth	LINC01123/SRSF7	Cancer-promoting	(33)
Colon cancer	*In vitro*	Cell proliferation, apoptosis, invasion, angiogenesis, chemosensitivity	LINC01123/miR-34c-5p/VEGFA	Cancer-promoting	(34)
Colorectal cancer	*In vitro* and *in vivo*	Cell viability, proliferation, apoptosis, migration, invasion, EMT, tumor growth	LINC01123/miR-625-5p/LASP1	Cancer-promoting	(42)
Ovarian cancer	*In vitro*	Cell proliferation, apoptosis, migration, invasion, EMT	LINC01123/miR-516b-5p/VEGFA	Cancer-promoting	(36)
Cervical cancer	*In vitro* and *in vivo*	Cell viability, migration, invasion, tumor growth	LINC01123/miR-361-3p/TSPAN1	Cancer-promoting	(37)
Osteosarcoma	*In vitro* and *in vivo*	Cell proliferation, apoptosis, migration, invasion, stemness, tumor growth	LINC01123/miR-516b-5p/Gli1, Hedgehog pathway	Cancer-promoting	(43)

A consistent finding across these studies is the elevated expression of LINC01123 in numerous tumor cell lines. This observation suggests that LINC01123 could serve as a potential biomarker for tumor aggressiveness, providing an avenue for further research into its prognostic value. At the subcellular level, LINC01123 predominantly localizes to the cytoplasm in six types of cancer cells: colon cancer (32, 34), breast cancer (39), HNSCC (27), LUAD (30, 40), NSCLC (28), glioma (29). This cytoplasmic localization is crucial, as it implicates LINC01123 in the regulation of cytoplasmic processes, including interaction with microRNAs and other cellular components.

**Figure 2:**
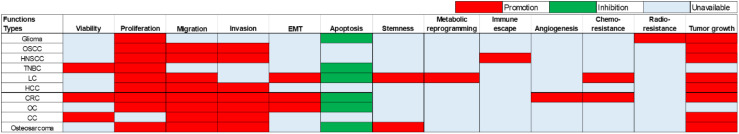
Diverse roles of oncogenic LINC01123 across ten different types of tumors. This underscores its wide-ranging impact in various cancers. These functions encompass the regulation of critical cellular processes such as proliferation, invasion, tumor growth, and more. OSCC, Oral Squamous Cell Carcinoma; HNSCC, Head and Neck Squamous Cell Carcinoma; TNBC, Triple-Negative Breast Cancer; LC, Lung Cancer; HCC, Hepatocellular Carcinoma; CRC, Colorectal Cancer; OC, Ovarian Cancer; CC, Cervical Cancer.

The oncogenic role of LINC01123 in tumorigenesis and development is multifaceted, influencing a variety of biological processes (see **Figure 2**). Notably, LINC01123 has been implicated in promoting EMT, which facilitates the metastatic capacity of tumors. By enhancing cell proliferation and inhibiting apoptosis, LINC01123 effectively increases cell viability, thereby supporting tumor growth. Moreover, LINC01123 facilitates not only cell migration and invasion but also induces a stem-like phenotype associated with tumor-initiating properties. This shift towards stemness suggests that LINC01123 may contribute to tumor recurrence and treatment resistance, as cancer stem cells are often implicated in these phenomena. Furthermore, LINC01123 promotes metabolic reprogramming and immune escape, thereby enabling tumors to thrive in hostile environments. This capability underscores the potential for targeted therapies that could disrupt LINC01123 interactions, rendering tumors more susceptible to immune responses and conventional treatments. Lastly, LINC01123’s involvement in angiogenesis and therapy resistance further highlights its critical role in sustaining tumor growth and resilience against treatment. Given these implications, LINC01123 emerges as a promising target for future therapeutic strategies aimed at combating a variety of cancers.

### 4.1 LINC01123 promotes tumor cell malignancy

LINC01123 has emerged as a pivotal regulator in various types of cancer, exerting significant influence on tumor cell malignancy through intricate molecular mechanisms.For example, in OSCC (26) and HCC (31), LINC01123 acts as a ceRNA by sequestering miR-34a-5p, which in turn upregulates TUFT1 expression. This regulatory interaction enhances cancer cell proliferation and invasion, highlighting LINC01123 as a potential therapeutic target to inhibit oncogenic processes. In TNBC (39), LINC01123 protects CMIP from miR-663a-mediated suppression. By acting as a miRNA sponge, LINC01123 promotes TNBC progression, implicating its role in modulating cellular processes crucial for tumor growth and metastasis. Studies in LUAD and CRC reveal that LINC01123 interacts with miR-4766-5p and miR-625-5p, respectively. In LUAD (30), LINC01123 regulates PYCR1 expression, influencing cancer cell proliferation and metastasis. Similarly, in CRC (42), LINC01123 competitively interacts with miR-625-5p to enhance LASP1 expression, thereby promoting tumor cell invasion and migration. In OC (36), LINC01123 regulates VEGFA through hsa-miR-516b-5p, impacting angiogenesis and tumor progression. Meanwhile, in CC (37), LINC01123 modulates the miR-361-3p/TSPAN1 axis to affect cell viability, migration, and invasion, underscoring its diverse roles in cancer cell behaviors. In osteosarcoma (43), LINC01123 promotes proliferation and metastasis via the miR-516b-5p/Gli1 axis, highlighting its oncogenic potential in bone cancer progression. Experimental evidence consistently shows that LINC01123 depletion inhibits tumor growth in preclinical models across these cancer types (30, 31, 33, 37, 42, 43). This underscores LINC01123 as a promising therapeutic target to disrupt oncogenic processes and improve patient outcomes. LINC01123 emerges as a critical regulator of tumor cell malignancy, exerting its effects through intricate ceRNA networks and signaling pathways. Understanding its multifaceted roles in promoting cancer progression provides a compelling rationale for further investigating targeted therapies aimed at suppressing LINC01123 activity in cancer cells.

### 4.2 LINC01123 regulates tumor metabolism and immunity

LINC01123 plays a critical role in shaping the tumor microenvironment through its dual regulation of metabolism and immunity in cancer (27, 28). Notably, in NSCLC, LINC01123 acts as a ceRNA, sequestering miR-199a-5p to enhance c-Myc expression (28). This interaction promotes metabolic adaptations, particularly increased glycolysis, which serves as a key energy source that fuels tumor growth and aggressiveness. The implication here is that by enhancing these metabolic pathways, LINC01123 not only supports tumor viability but also positions tumors to thrive under metabolic stress, further exacerbating their malignancy.

Moreover, LINC01123 establishes a positive feedback loop with c-Myc, which further amplifies these metabolic changes and facilitates tumor progression and metabolic reprogramming (28). This relationship highlights the potential for targeting this loop therapeutically; disrupting the feedback mechanism could impair tumor growth and shift metabolic reliance, rendering cancers more vulnerable to treatment.

Beyond its metabolic implications, LINC01123 significantly influences tumor immunity (27). In HNSCC, LINC01123 interacts with B7-H3 (27), a protein intricately linked to both immune escape and non-immune pathways of tumor invasion (44–46). The consequences of this interaction are profound; LINC01123 overexpression or the downregulation of miR-214-3p in HNSCC cells leads to dysfunctional CD8+ T cells (27). This dysfunction is characterized by decreased expression levels of key immune markers such as TNF-α, IFN-γ, perforin, and granzyme B (27). These markers are crucial for effective CD8+ T cell-mediated immune responses against tumors (47–49). The reversibility of LINC01123’s detrimental effects through miR-214-3p silencing suggests promising therapeutic strategies aimed at restoring immune function and enhancing anti-tumor responses in HNSCC (27). By re-establishing the balance in this regulatory axis, it may be feasible to reinvigorate the immune response, offering a potential avenue for improving treatment outcomes.

Overall, understanding the intricate roles of LINC01123 in both metabolic reprogramming and immune evasion underscores its significance as a target in the development of novel therapies. By addressing both the metabolic and immune facets of tumor biology, strategies targeting LINC01123 could yield substantial improvements in combating tumor progression and enhancing the efficacy of existing treatments in cancer.

### 4.3 LINC01123 enhances treatment resistance

LINC01123 exhibits a crucial role in promoting resistance to treatment across different types of cancer. In glioma, LINC01123 is significantly upregulated in radioresistant cells (U251R), where it acts as a ceRNA by binding to miR-151a (29). This interaction decreases miR-151a levels, resulting in increased expression of CENPB. Elevated CENPB expression contributes to radioresistance by facilitating DNA repair mechanisms, as validated through *in vitro* and *in vivo* experiments involving xenograft tumors exposed to irradiation (29). In LUAD, LINC01123 is implicated in mediating cisplatin resistance through ceRNA pathways involving other lncRNAs such as HOXD-AS2 and FIRRE (41). This suggests a broader role for LINC01123 in modulating chemotherapy resistance mechanisms. In colon cancer, LINC01123 localizes predominantly in the cytoplasm, where it competes with VEGFA for binding to miR-34c-5p (34). By sequestering miR-34c-5p, LINC01123 upregulates VEGFA expression at both mRNA and protein levels, promoting angiogenesis and potentially contributing to chemoresistance (34). LINC01123 plays a pivotal role in enhancing resistance to treatment in various cancers by regulating ceRNA networks and influencing key downstream effectors. Targeting LINC01123 and its ceRNA networks could potentially reverse treatment resistance.

## 5 Mechanisms of LINC01123 in regulating biological functions

### 5.1 Regulation through ceRNA networks

**Figure 3:**
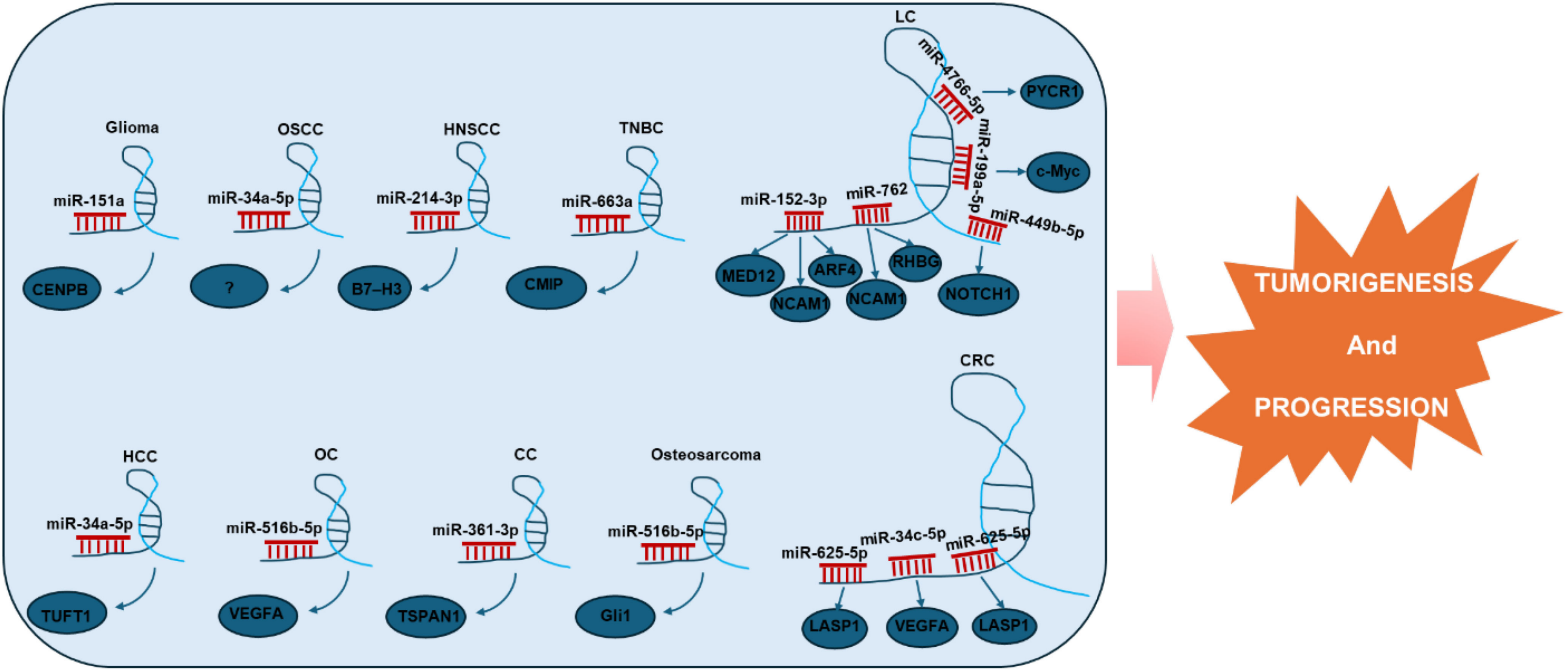
LINC01123 participates in a complex ceRNA network, competitively binding with miRNAs in various cancers. This interaction regulates the downregulation of oncogenic targets, thereby promoting tumorigenesis and disease progression. OSCC, Oral Squamous Cell Carcinoma; HNSCC, Head and Neck Squamous Cell Carcinoma; TNBC, Triple-Negative Breast Cancer; LC, Lung Cancer; HCC, Hepatocellular Carcinoma; CRC, Colorectal Cancer; OC, Ovarian Cancer; CC, Cervical Cancer.

The role of LINC01123 as a ceRNA has been comprehensively studied across a variety of tumors (26–32, 34, 36, 37, 39–43), revealing diverse mechanisms through which it influences tumor biology. For instance, in glioma, LINC01123 has been shown to regulate the miR-151a/CENPB pathway, thereby enhancing radioresistance (29). This suggests that targeting LINC01123 could potentially improve therapeutic outcomes in glioma treatment by overcoming resistance. Similarly, in OSCC, LINC01123 promotes tumor progression by sponging miR-34a-5p (26). The implication here is significant; by inhibiting a key microRNA associated with tumor suppression, LINC01123 may facilitate a more aggressive tumor phenotype, highlighting the need for strategies that disrupt this interaction. In HNSCC, LINC01123 regulates the expression of B7-H3 through sponging miR-214-3p. This interaction leads to B7-H3 upregulation, which inhibits CD8+ T cell activation and contributes to immune evasion (27). Consequently, targeting this pathway may enhance immunotherapeutic efficacy against HNSCC by reinstating T cell activation. In TNBC, LINC01123 exerts influence over tumor growth via the LINC01123/miR-663a/CMIP axis (39). This suggests a potential therapeutic avenue; by disrupting this axis, it may be possible to mitigate tumor growth and progression in TNBC. In LUAD, Li et al. (41) demonstrated that LINC01123 could contribute to cisplatin resistance. This is achieved through ceRNA networks involving hsa-miR-152-3p/MED12-NCAM1-ARF4 and hsa-miR-762/NCAM1-RHBG axes. These findings stress the importance of LINC01123 in chemoresistance, suggesting that targeting it could enhance the effectiveness of cisplatin in LUAD therapy. Additionally, both *in vitro* and *in vivo* studies have shown that LINC01123 accelerates malignancy in NSCLC by acting as a ceRNA, thereby influencing multiple axes including miR-4766-5p/PYCR1 (30), miR-199a-5p/c-Myc (28), and miR-449b-5p/NOTCH1 (40). These findings highlight the multifaceted role of LINC01123 in promoting NSCLC, reinforcing the notion of its potential as a target for therapeutic intervention. In HCC, it promotes tumor cell proliferation and invasion by modulating the miR-34a-5p/TUFT1 axis (31). This suggests that LINC01123 could be a valuable marker for HCC aggressiveness. In CRC, LINC01123 drives tumor progression by regulating miR-34c-5p/VEGFA (34) and miR-625-5p/LASP1 axes (32, 42). These interactions underline the potential of LINC01123 as a therapeutic target to impede CRC advancement. Furthermore, in ovarian cancer, LINC01123 enhances malignancy by competitively binding to hsa-miR-516b-5p, leading to VEGFA upregulation (36). This pathway suggests a critical role for LINC01123 in fostering an angiogenic tumor environment. In cervical cancer, LINC01123 stimulates proliferation, migration, and invasion by inhibiting miR-361-3p and upregulating TSPAN1 (37). This interplay underscores the necessity of investigating LINC01123’s mechanisms in cervical cancer to potentially hinder its metastatic capabilities. Lastly, LINC01123 enhances osteosarcoma proliferation and metastasis via the miR-516b-5p/Gli1 axis (43). This highlights the potential for LINC01123 to serve as a therapeutic target across various cancer types. All LINC01123-associated ceRNA networks are illustrated in **Figure 3**.

### 5.2 Formation of RNA-protein complexes

The primary function of lncRNAs involves their interaction with cellular macromolecules, which is crucial for various regulatory processes within the cell (50–52). Specifically, LINC01123 is capable of forming RNA-protein complexes, utilizing specific proteins as structural components. This interaction not only facilitates the modulation of LINC01123 but also significantly affects the expression and functionality of subsequent molecular targets.

In CRC, Liu et al. (33) provided compelling evidence that LINC01123 specifically interacts with SRSF7, a notable member of the splicing factor family involved in critical cellular processes such as splicing, transport, and polypeptide synthesis (53–55). The expression levels of LINC01123 positively correlate with those of SRSF7, indicating that higher levels of LINC01123 may enhance the activity of SRSF7. SRSF7 has been found to be overexpressed in various cancers and plays a significant role in regulating the alternative splicing of key oncogenes (56–59). This raises important implications: by forming RNA-protein complexes with SRSF7, LINC01123 may alter alternative splicing processes, potentially leading to the upregulation of downstream oncogenes that drive tumorigenesis.

The functional consequences of this interaction are substantial; experimental studies have demonstrated that through these RNA-protein complexes, LINC01123 not only influences splicing but also promotes tumor growth and migration (33). Such effects have been validated in both *in vitro* and *in vivo* experiments (33), indicating a potential pathway through which LINC01123 contributes to CRC progression and underscores its role as a critical factor in cancer biology.

## 6 Future perspectives

A huge amount of evidence recent suggested that lncRNAs play a vital role in the development of human diseases (60–62). Further investigation into different lncRNAs in human cancers, particularly their involvement in both the onset and advancement of tumors, is valuable and warrants exploration and consolidation. Here, we comprehensively examine current research the clinical values and roles of LINC01123 in human tumors and provides insights into its molecular regulatory mechanisms.

LINC01123, a newly identified lncRNA, consistently exhibits upregulation in various cancerous tissues and cell lines (26–38), suggesting its potential as an oncogene in tumorigenesis. Similarly, overexpression of LINC01089 is associated with advanced clinicopathological features across different cancer types (26, 28, 31, 32, 37), including larger tumor size, lymph node metastasis, and higher TNM stage. Elevated levels of LINC01089 also correlate with poorer prognosis (26–28, 31–33, 38), leading to shorter overall survival and accelerated cancer progression. Thus, LINC01123 serve as a promising prognostic biomarker in multiple tumors.

**Figure 4:**
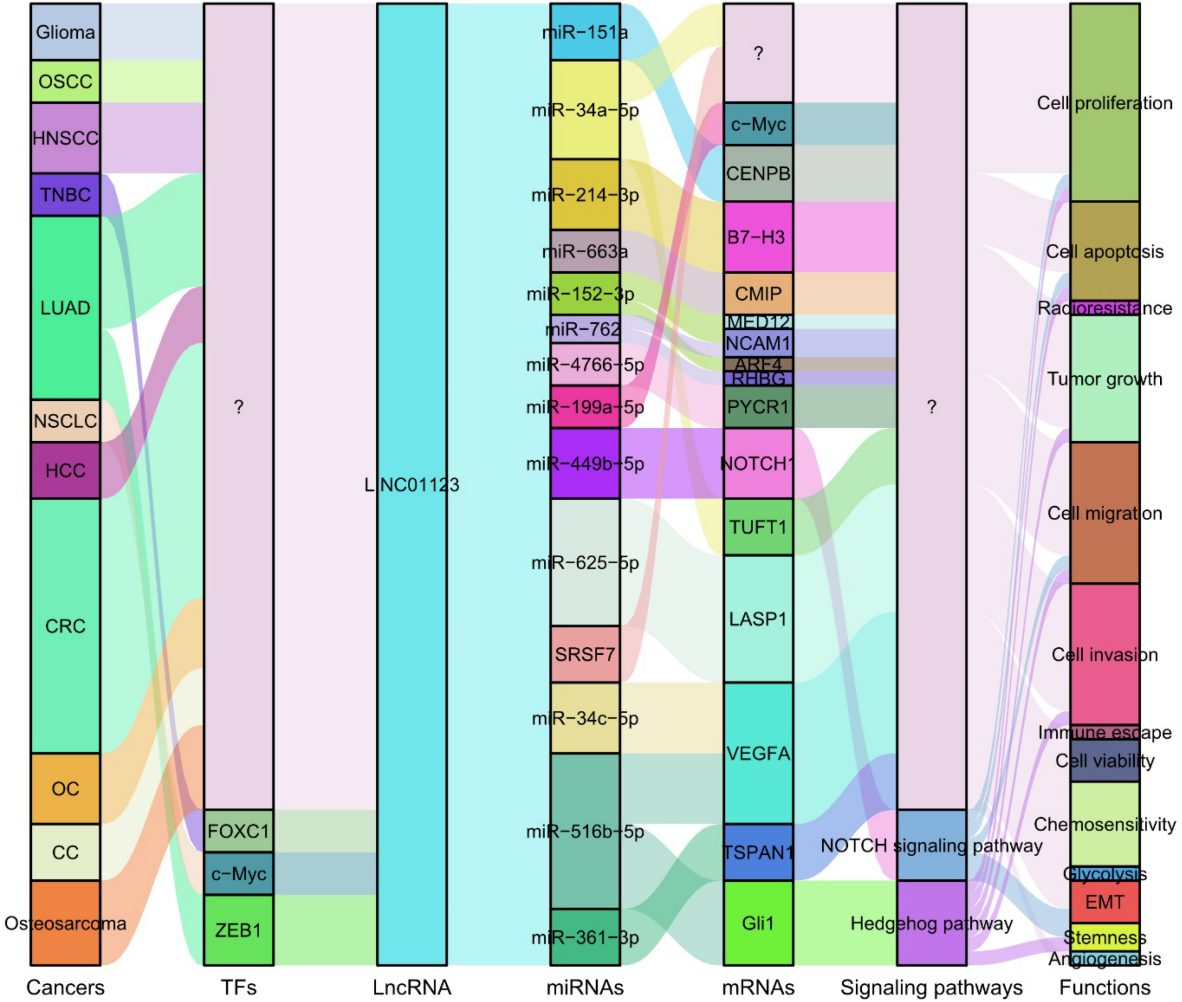
Summary of the mechanisms by which LINC01123 participates in various biological processes associated with tumor pathogenesis and progression. LINC01123 is regulated by key transcription factors, including FOXC1, c-Myc, and ZEB1. LINC01123 participates in multiple lncRNA-miRNA-mRNA ceRNA networks and regulates critical signaling pathways such as NOTCH and Hedgehog. Through these mechanisms, LINC01123 functions as a pivotal oncogenic factor, modulating various tumor processes. OSCC, Oral Squamous Cell Carcinoma; HNSCC, Head and Neck Squamous Cell Carcinoma; TNBC, Triple-Negative Breast Cancer; LUAD, Lung Adenocarcinoma; NSCLC, Non-Small Cell Lung Cancer; HCC, Hepatocellular Carcinoma; CRC, Colorectal Cancer; OC, Ovarian Cancer; CC, Cervical Cancer; TFs, Transcription Factors; EMT, Epithelial-Mesenchymal Transition.

Recent experimental investigations have characterized the functional roles of LINC01123 across eleven distinct cancer types (**Table 2**), including glioma (29), OSCC (27), HNSCC (27), TNBC (39), LUAD (30, 40, 41), NSCLC (28), HCC (31), CRC (32–34, 42), OC (36), CC (37) and osteosarcoma (43). These studies have elucidated its regulatory interactions with critical molecular targets, key signaling pathways, and essential biological processes that drive tumorigenesis and cancer progression, as illustrated in **Figure 4**. LINC01123 functions as a pivotal oncogenic factor by regulating tumor cell proliferation, apoptosis, viability, invasion, migration, and EMT. Notably, LINC01123 is also implicated in stemness, glycolysis, angiogenesis, and resistance to various tumor therapies. These effects are mediated through diverse mechanisms, including the lncRNA-miRNA-mRNA ceRNA network, NOTCH and Hedgehog pathways, and interactions with SRSF7. Consequently, LINC01123 plays critical roles in tumor progression, glycolysis, tumor immunity, and therapy resistance. Targeting LINC01123 has thus emerged as a promising therapeutic strategy for cancer treatment.

Research on LINC01123 indicates its expression has been studied in nine types of solid tumors (**Table 1**), including glioma (29), lung cancer (LUAD, NSCLC) (28, 30), OSCC (26), HNSCC (27), HCC (31), CRC (32–34), intrahepatic cholangiocarcinoma (35), OC (36) and CC (37), yet its presence in hematologic malignancies and other solid cancers remains unexplored. To establish its broader relevance, future studies should investigate its expression across hematologic malignancies and additional solid cancer types. Moreover, while LINC01123 has shown prognostic implications in seven tumor types (26–28, 31–33, 38), its clinical significance across diverse cancer stages and types requires validation through larger patient cohorts. On the other hand, LINC01123 has been implicated in various tumors, indicating a need for further *in vivo* and *in vitro* studies to delineate its specific roles and mechanisms in different cancer contexts. Its complex functions underscore the importance of gaining comprehensive insights into its involvement in distinct pathways and ceRNA networks, as well as lncRNA-protein interactions across different cancers. Additionally, studies have explored its potential associations with tumor metabolism in NSCLC (28) and immunity in HNSCC (27), while research into drug resistance is currently limited to radioresistance in glioma (29), chemotherapy resistance in LUAD (41), and colon cancer (34). Further studies are needed to explore these associations in more depth, mechanistically and across more cancer types.

## 7 Conclusion

In conclusion, LINC01123 has emerged as a newly identified and dysregulated cancer-associated lncRNA, highlighting its potential as both a promising biomarker and a key player in multiple critical aspects of tumor progression, metabolism, immune evasion, and therapeutic resistance. Targeting LINC01123 may offer new therapeutic avenues and enhance the efficacy of existing treatment options. However, further mechanistic investigations and large-scale comprehensive clinical studies are essential to elucidate its precise role in human cancer pathogenesis and to assess its clinical applicability.
